# Visual Performance of Individuals With and Without Meibomian Gland Dysfunction

**DOI:** 10.7759/cureus.81655

**Published:** 2025-04-03

**Authors:** Isabella Tunon-Robinson, Jingyu Huang, Xiaoming Xu, Ashley K Nguyen

**Affiliations:** 1 School of Computing and Augmented Intelligence, Arizona State University, Tempe, USA; 2 Statistics, Allergan, an AbbVie company, Irvine, USA; 3 Clinical Development, Allergan, an AbbVie company, Irvine, USA

**Keywords:** disease severity, dry eye disease, meibomian gland dysfunction, quality of life, reading speed, visual acuity, visual function

## Abstract

Purpose

Dry eye disease (DED) is associated with decreased visual function, such as deterioration in reading speed. Changes in visual function in individuals with meibomian gland dysfunction (MGD), which is commonly associated with evaporative DED, have not been well studied. This study evaluated best-corrected visual acuity (BCVA) and reading speed in individuals with and without MGD.

Methods

In a 22-day, prospective, noninterventional, multicenter (three sites) clinical study (NCT01979887), eligible adults (age ≥40 years with no uncontrolled ocular or systemic disease other than MGD) were classified into one of three cohorts (non-MGD, mild/moderate MGD, and severe MGD) based on meibum quality scores, ocular symptom scores, and Schirmer test results. Visual function tests included BCVA in the study eye and reading speed evaluated using the International Reading Speed Texts (IReST) tool with both eyes refracted for best correction at 40 cm. The average of measurements from days one and 22 was used for analysis, and nominal p values were reported.

Results

Seventy-five individuals were assigned to the cohorts (25 per cohort). Overall, the mean age of the subjects was 54.5 years, and 66.7% (50/75) were women. Mean Schirmer (without anesthesia) test scores in the study eye ranged from 16.4 to 18.6 mm among cohorts, indicating that, on average, the subjects were not aqueous deficient. Mean BCVA was 89.9, 88.3, and 82.7 letters in the non-MGD, mild/moderate MGD, and severe MGD cohorts, respectively (p= 0.046 severe vs mild/moderate MGD; p= 0.094 severe MGD vs non-MGD). Mean IReST reading speed was slower in the severe MGD cohort (146.0 words/min) than in the non-MGD (164.2 words/min) and mild/moderate MGD (167.8 words/min) cohorts, and the difference between the mild/moderate and severe MGD cohorts was significant (p= 0.046).

Conclusions

Tear film stability is important for the maintenance of visual performance. Both BCVA and reading speed were reduced in patients with severe MGD. For patients with MGD (as defined in this protocol) and visual complaints, reading speed testing in the office may be beneficial to detect patients’ difficulty with visual tasks in everyday life.

## Introduction

Dry eye disease (DED) is a prevalent disease of the ocular surface characterized by loss of homeostasis of the tear film and accompanied by ocular symptoms, such as ocular discomfort and visual disturbance [1]. Common visual complaints include blurred vision, fluctuating vision with blinking, vision, glare, and eye fatigue, which can fluctuate in severity [2]. DED can be classified as aqueous-deficient, evaporative, or mixed [1]. The most common cause of evaporative DED is meibomian gland dysfunction (MGD) [3]. The meibomian glands in the lids secrete meibum, which spreads onto the ocular surface and forms the outer layer of the tear film, the tear film lipid layer (TFLL). The TFLL helps tears spread to lubricate the ocular surface, serves as a barrier to evaporation, and stabilizes the tear film by reducing surface tension [4,5]. In MGD, the quantity and quality of the meibum are reduced, resulting in changes in the TFLL that cause tear film instability [6].

DED is associated with decreased vision-related quality of life (QoL). Patients with DED often report difficulty with visual tasks in daily living that require sustained gazing, such as reading a newspaper, driving, or working on a computer [7]. In a recent large population-based study (89,022 participants), individuals with DED reported reduced vision-related QoL in all 10 domains (general health, general vision, near activities, distance activities, peripheral vision, color vision, social functioning, driving, worrying about eyesight, and ocular pain), with the risk of reduced score on each domain comparable to or greater than that in individuals with macular degeneration [8].

Visual acuity measurements frequently show excellent visual acuity in patients with DED. Most studies using standard visual acuity testing have not shown differences between subjects with DED and normal control subjects, because standard visual acuity testing is able to detect visual disturbances only in severe cases of DED [2]. In less severe cases, individuals may be able to increase the frequency of blinking to compensate for an inadequate tear film during the examination. Dynamic visual acuity testing [9], measurements of contrast sensitivity [7], and post-blink blur time [10] (the time until best-corrected visual acuity decreases after a blink) are more sensitive to tear film instability and may be better able to detect problems with visual performance in patients with DED. Interestingly, accommodative microfluctuations have been shown to be increased in patients with a short tear break-up time (TBUT) and symptoms of DED [11]. This increase in accommodative microfluctuations could potentially be a compensatory mechanism to help maintain accommodation and visual function when tear film instability causes blurry vision [12].

Reading tests have been able to detect changes in visual function that impact QoL in patients with DED in a number of clinical studies [7,13,14]; however, these studies did not specifically study patients with MGD. In one of these studies, the reading speed of subjects with and without DED was evaluated on a variety of reading tests, and those with DED had slower reading speeds on each test [13]. The International Reading Speed Texts [15] (IReST) are paragraphs on a sixth-grade reading level that are available in 19 languages and read aloud in reading speed tests. The number of words read correctly per minute (wpm) is a standardized and reproducible measurement of reading speed. Mathews et al. [14] reported a comparison study of IReST reading speed in subjects with DED and normal control subjects. There was no difference between groups in the mean (habitual corrected) visual acuity in the better eye, but the mean reading speed on an IReST text was significantly slower in the DED group (148 vs 163 wpm, p = 0.006). Subsequently, a study by Akpek et al. [7] showed that iReST reading speed decreased from 172 to 161 wpm (p = 0.02) after a silent reading task requiring sustaining gazing in subjects with DED, while there was no decrease in reading speed in control subjects. There was an association between the decrease in reading speed and corneal staining, indicating a relationship between corneal damage and functional vision loss.

Although visual function effects have been studied in DED, clinical studies on the alterations in visual function associated with MGD are lacking. The approach to treating patients with MGD and evaporative DED versus patients with aqueous tear deficiency can be very different [16,17]. A basic understanding of the differential effects of these two types of DED on visual function is important, especially since these outcomes can be used as registration endpoints in clinical trials for approval of drug products to treat DED [18].

A previously reported 22-day, prospective, observational clinical study evaluated signs and symptoms [19]; changes in meibum composition [20,21], meibomian gland structure [22], and TFLL thickness [23]; and changes in QoL [24] associated with the presence and severity of MGD. The aim of the present analysis was to evaluate best-corrected visual acuity (BCVA) and IReST reading speed in individuals with and without MGD in the study. 

This work was previously presented in part in a poster at the Association for Research in Vision and Ophthalmology (ARVO) 2025 Annual Meeting, May 4-8, 2025, Salt Lake City, Utah.

## Materials and methods

This exploratory, 22-day, prospective, noninterventional, clinical study was performed to investigate signs and symptoms associated with the presence and severity of MGD. The study was conducted at two sites in the United States and one site in England in accordance with the principles of the Declaration of Helsinki, and all participants provided written informed consent. The study was registered at ClinicalTrials.gov with the identifier NCT01979887.

The study design and methods were published previously [19,21-24]. Briefly, the study enrolled adults aged 40 years or older with no uncontrolled systemic disease and no uncontrolled ocular disease other than MGD. All study participants underwent ophthalmic examinations for signs and symptoms of MGD at the first (day one) study visit. Based on the results of the examinations, study participants who qualified were assigned to one of three study cohorts (non-MGD, mild/moderate MGD, and severe MGD) until the planned sample size of 25 individuals per cohort was achieved. The qualification criteria for cohort assignment (Table 1) were consistent with diagnostic and severity grading guidelines from the Tear Film and Ocular Surface Society (TFOS) International Workshop on Meibomian Gland Dysfunction [19,25] and included the maximum meibum quality score (MMQS) obtained in the evaluation of meibum from six central meibomian glands in the lower lid, the sum of scores for the worst two symptoms on an ocular symptom questionnaire, and Schirmer test results.

**Table 1:** Cohort assignment criteria Adapted from Ajouz et al. [19]. ^a^The meibum quality of six central meibomian glands in the lower lid was graded on a scale of 0 = clear excreta or clear with small particles (normal viscosity); 1 = opaque excreta with normal viscosity; 2 = opaque excreta with increased viscosity (gel-like); 3 = secretions retain shape, or secretions do not completely express but a toothpaste-like substance can be seen at the opening of the orifice; and NE = nonexpressible (nothing at orifice or metaplastic). MGD, meibomian gland dysfunction; MMQS, maximum meibum quality score (among all six graded glands)

Cohort	Investigator-graded MMQS in Study Eye^a^	Schirmer Tear Test Without Anesthesia in Study Eye	Sum of Scores of the Worst Two Symptoms on the Ocular Symptom Questionnaire
Non-MGD	0 or 1	≥7 mm/5 min	0 to 4 with neither symptom scored as >2
Mild/moderate MGD	2	≥7 mm/5 min	0 to 4 with neither symptom scored as >2
Severe MGD	3	≥7 mm/5 min	≥4

For an individual to qualify for cohort assignment, at least one eye (designated as the study eye) had to meet the criteria associated with the cohort. If the individual’s eyes met the criteria for different cohorts, the eye with the higher MMQS was designated as the study eye, and the individual was assigned to the corresponding cohort. The study participants who were assigned to a cohort were reexamined at a second study visit on day 22 (the exit visit). Those who were not assigned to a cohort were discontinued from the study.

Ocular symptoms, meibum expressibility and the quality of secretions, TBUT, lissamine green staining of the upper and lower lid margins, meibography and biomicroscopy of the upper and lower lids, and participant responses on the MGD Impact Questionnaire were evaluated at both study visits. Schirmer tear tests without anesthesia and corneal staining with sodium fluorescein were evaluated at the day one visit only. TFLL thickness was assessed with an ocular surface interferometer at a single site at both study visits. Meibum samples were collected at the exit visit and analyzed with spectroscopy to identify changes in meibum composition associated with the presence and severity of MGD. The results of these assessments were reported previously [19-24].

Visual function was evaluated with tests of BCVA and reading speed at both study visits. BCVA with manifest refraction was measured in each eye on a logMAR chart at a 3 m distance. The lowest line read with one or no misses was determined to be the BCVA and recorded in Snellen equivalent units.

Reading speed was evaluated using the IReST instrument [15] with both eyes refracted for best correction at 40 cm. Participants were administered the test in English or Spanish, as appropriate. Reported standard scores, mean ± standard deviation (SD), for the English and Spanish tests, are 228 ± 30 wpm and 218 ± 28 wpm, respectively [15]. Testing in English used IReST text number 3 at the enrollment visit and text number 5 at the exit visit. Testing in Spanish used IReST text numbers 4 and 6 at the enrollment and exit visits, respectively. At each test administration, the participant was asked to read the entire text (approximately 130 words) aloud as quickly as possible, without going back and making corrections. At the start of the test, the participant uncovered the text and began to read aloud, and the time to read the entire text was measured with a stopwatch and recorded. The number of words misread or missed was also recorded and used in the calculation of reading speed.

Statistical analysis

Snellen BCVA values were converted to Early Treatment Diabetic Retinopathy Score (ETDRS) letters for analysis [26]. BCVA in the study eye of each participant was averaged over the day one and day 22 visits and analyzed for pairwise comparisons between cohorts using an analysis of variance (ANOVA) model with factors of cohort and site.

Reading speed in wpm was calculated with the following formula: reading speed (wpm) = (60/reading time in seconds) x (number of correctly read words in the passage). The calculated reading speed for each participant was averaged over the day one and day 22 visits and analyzed for pairwise comparisons between cohorts using an ANOVA model with factors of cohort and site.

As this study was exploratory, all statistical comparisons were made at the α = 0.05 level without adjustment for multiple comparisons. Nominal p values are reported.

## Results

Seventy-five individuals were assigned to the cohorts (25 per cohort). Table 2 lists demographic and study eye clinical characteristics in each cohort. Overall, the mean subject age was 54.5 years, 66.7% (50/75) were women, 30.7% (23/75) were Caucasian, and 44.0% (33/75) were Black. The proportion of Black and Hispanic subjects was higher in the mild/moderate and severe MGD cohorts than in the non-MGD cohort (Table 2). Mean Schirmer (without anesthesia) test scores in the study eye ranged from 16.4 to 18.6 mm among the cohorts, indicating that, on average, the subjects were not aqueous deficient.

**Table 2:** Demographics and study eye clinical characteristics in the cohorts MGD, meibomian gland dysfunction; MMQS, maximum meibum quality score (among all six graded glands on the central lower lid); SD, standard deviation Note: The authors note that the subjects were not asked about the countries of their ancestors’ origin; hence, the terms "Caucasian," "Black," "Asian," and "Hispanic" are retained for accuracy.

Parameter	Non-MGD (n = 25)	Mild/moderate MGD (n = 25)	Severe MGD (n = 25)
Age, mean (SD), years	52.0 (8.34)	52.8 (6.26)	58.8 (11.86)
Range	40–74	43–63	41–89
Sex (self-identified), n (%)			
Male	9 (36)	9 (36)	7 (28)
Female	16 (64)	16 (64)	18 (72)
Race (self-identified), n (%)			
Caucasian	13 (52)	6 (24)	4 (16)
Black	7 (28)	15 (60)	11 (44)
Asian	1 (4)	1 (4)	2 (8)
Hispanic	1 (4)	2 (8)	8 (32)
Other	3 (12)	1 (4)	0
Schirmer test score, mean (range), mm	16.4 (7–35)	18.6 (7–35)	16.7 (7–40)
MMQS, mean (SD)	0.3 (0.48)	2 (0)	3 (0)

**Figure 1:**
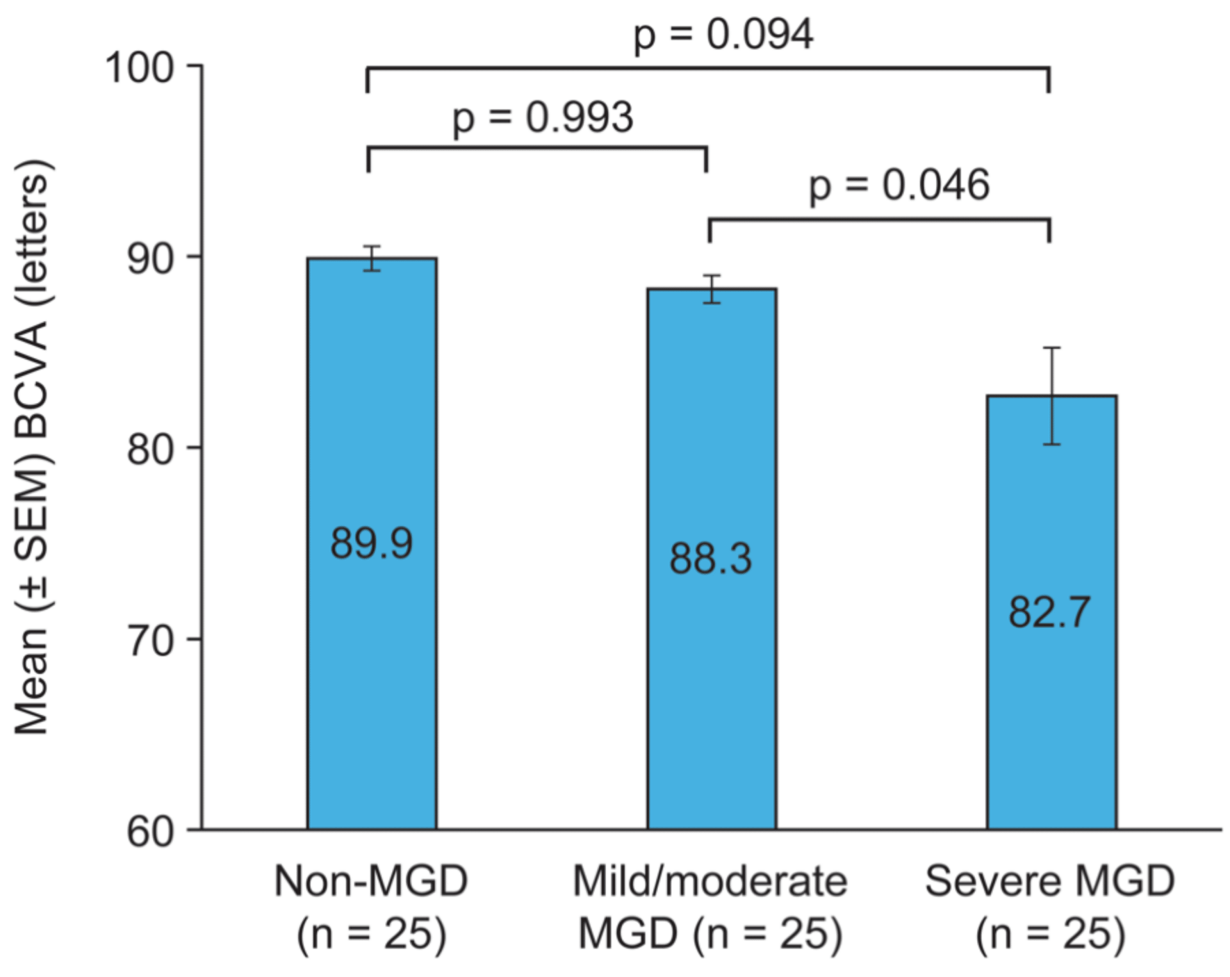
Mean BCVA in the cohorts For each subject, the average of BCVA measurements in the study eye at the day one (enrollment) and day 22 (exit) visits was used for analysis. BCVA, best-corrected visual acuity; MGD, meibomian gland dysfunction; SEM, standard error of the mean

Mean BCVA was worse in the severe MGD cohort compared with the non-MGD and mild/moderate MGD cohorts (Figure 1). The mean (± SD) BCVA was 89.9 ± 3.19, 88.3 ± 3.59, and 82.7 ± 12.68 letters in the non-MGD, mild/moderate MGD, and severe MGD cohorts, respectively. The difference in BCVA between severe and mild/moderate MGD was significant (p = 0.046). 

**Figure 2:**
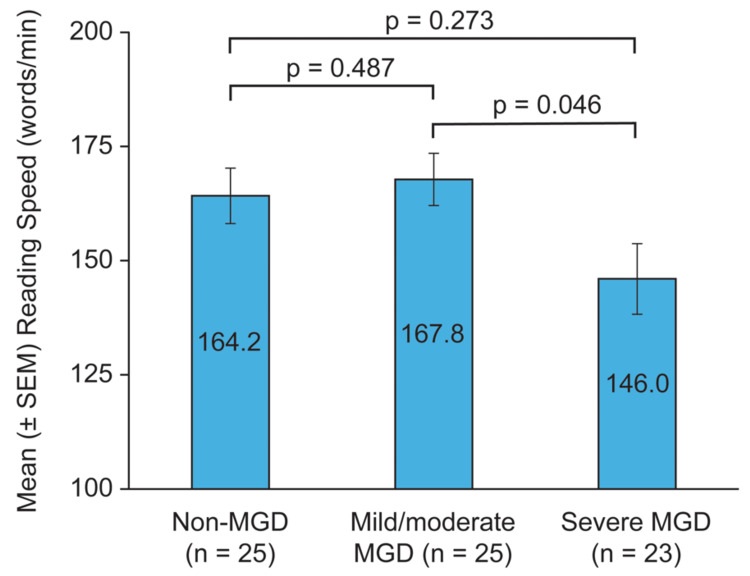
Mean reading speed in the cohorts For each subject, the average of reading speed measurements at the day one (enrollment) and day 22 (exit) visits were used for analysis. MGD, meibomian gland dysfunction; SEM, standard error of the mean

The mean IReST reading speed was slower in the severe MGD cohort than in the non-MGD and mild/moderate MGD cohorts (Figure 2). The mean (± SD) reading speed was 164.2 ± 30.36, 167.8 ± 28.56, and 146.0 ± 37.06 words/min in the non-MGD, mild/moderate MGD, and severe MGD cohorts, respectively. The difference in reading speed between the mild/moderate and severe MGD cohorts was significant (p = 0.046). 

## Discussion

This study demonstrated a reduction in BCVA in individuals with severe MGD. The difference in mean BCVA between the severe MGD cohort and the non-MGD and mild/moderate MGD cohorts was >5 ETDRS letters (one line). The mean BCVA was excellent (better than 20/20 Snellen equivalent) in the non-MGD and mild/moderate cohorts and very good (20/22 Snellen equivalent) in the severe MGD cohort. The BCVA analysis was based on the study eye, which met rigorous standards for meibum quality, symptom score, and Schirmer score required for assignment to the non-MGD, mild/moderate MGD, or severe MGD cohort.

Reading speed was also reduced in individuals with severe MGD. The mean reading speed was approximately 20 wpm slower in the severe MGD cohort compared with the non-MGD and mild/moderate MGD cohorts. The slower reading speed in the severe MGD cohort is considered clinically significant, as previous studies have considered IReST reading rate differences of >10 wpm to be clinically relevant [15]. The observed reading speed in the non-MGD cohort (164.2 wpm) was slower than the published standard of 228 wpm for the English IReST [15]. However, this standard was based on data from a different demographic than is typically seen in DED and MGD, since much younger subjects aged 18-35 years with normal vision participated in the standardization study [15]. However, the reading speed in the non-MGD group was almost identical to the IReST reading speed of 163 wpm observed in the control group of a study comparing reading speeds in subjects with and without DED [14] that enrolled subjects with a similar demographic as in our study. The reasons for the slower-than-standard reading speed in the non-MGD cohort in our study are not well understood. However, the mean age in the cohort was 52 years, and there is a known effect of age on IReST reading speed, with older age associated with slower reading speed [27,28]. The educational level of subjects also has been shown to affect IReST reading speed, but the effect lessens at older ages [28].

We did not observe a difference in reading speed between the non-MGD and the mild/moderate MGD cohort. This implies that the severity of MGD has to meet a critical threshold before affecting visual function. In a clinical trial for drug registration, it is critical to be able to show a difference in outcome measures between the drug-treated and placebo-treated arms [18]. If patients with mild/moderate MGD are enrolled and their baseline is similar to what is expected in individuals without MGD, there is no room to show improvement in reading speed because of a ceiling effect [29] that reduces the sensitivity of the reading speed instrument to detect a change. If functional endpoints such as reading speed are employed, our suggestion would be to enroll patients with a severity profile similar to that in our severe MGD cohort, who have significantly slower reading speeds at baseline, to enrich the population that would be able to see a treatment benefit, if one exists, between the drug-treated and placebo-treated arms.

Results of the BCVA and reading speed assessments were consistent with the study participants’ assessments of their QoL on the novel MGD Impact Questionnaire [24]. The severe MGD cohort reported greater difficulty with reading, driving, and performing leisure activities; more frequent difficulty with outdoor activities; more time spent on eye care; and greater bother with time spent on eye care than the non-MGD cohort [24]. In comparison with the mild/moderate MGD cohort, the severe MGD cohort reported significantly greater difficulty with reading and performance of leisure activities, more time spent on eye care, and greater bother with eye appearance and time spent on eye care [24]. Higher-order aberrations and light scattering caused by tear film instability are believed to cause vision complaints and loss of visual quality and function in DED [2,30]. Standard visual acuity testing is relatively insensitive to these alterations in optics. BCVA measurements in this study were able to differentiate the severe MGD cohort from the non-MGD and mild/moderate MGD cohorts, and while measurements in the severe MGD cohort showed greater variability, the mean visual acuity was still good. The visual acuity measurement methods in our clinical trial used a logMAR chart. However, in the clinical setting outside of a clinical trial, visual acuity measurements typically are performed using less precise Snellen eye charts, which measure visual acuity to the nearest line read accurately, but not the nearest letter. Therefore, it would be difficult to identify the presence of functional vision disturbances related to severe MGD based on visual acuity in a typical clinical setting. In contrast, a large decline in reading speed was observed in the severe MGD cohort, suggesting that reading tests administered in the clinical setting may better indicate whether a patient is having difficulties with vision. Furthermore, unlike reading individual letters on a vision chart, reading IReST passages is similar to activities of daily living such as reading a newspaper, and IReST reading speed results may reflect the patient’s visual complaints and difficulties with real-life visual tasks. Given reading speed’s positive attribute of reflecting effects on activities of daily living, the IReST instrument was employed in a recent clinical trial of a device delivering heat therapy to the eyelids to help evaluate the device’s effectiveness in improving outcomes in patients with MGD and DED [31].

The observed decrease in visual performance in the severe MGD cohort is consistent with the previously reported differences in tear film stability among the cohorts [21]. Mean TBUT scores at the exit visit followed the trend of non-MGD > mild/moderate MGD > severe MGD, with TBUT significantly lower in the severe MGD cohort than in the non-MGD cohort [21]. No significant differences in TFLL thickness were noted among the cohorts [23], but meibum composition was altered in the MGD cohorts compared with the non-MGD cohort, with the largest changes seen in the severe MGD cohort [20,21]. Further, the area of meibomian gland dropout was associated with meibum quality and was largest in the severe MGD cohort [22]. The mean area of meibomian gland dropout in the lower lid on day one (Arias 0-3 scale) was 0.42 in the non-MGD group, 0.72 in the mild/moderate MGD group, and 1.28 in the severe MGD group [22]. Consistent with the findings of other studies in MGD, the upper lid was less affected by gland dropout [22]. Overall, these results suggest that changes in the lipid layer of the tear film in MGD leading to tear film instability may be related more to the quality of the lipid than to the quantity [23].

This study has limitations that are common in exploratory studies, including a small sample size and a limited number of study sites. Nevertheless, the results and conclusions are directional and can inform larger clinical studies interested in visual performance difficulties in patients with MGD. In addition, there was an imbalance in race/ethnicity across the study cohorts, which could potentially reflect a greater prevalence of MGD in Black and Hispanic individuals, but potential differences in the prevalence of MGD across races and ethnicities require further study [32]. Finally, as previously stated, DED can be classified as aqueous-deficient, evaporative, or mixed, in which there is a contribution of both aqueous deficiency and evaporative dry eye. Our study objectives were focused on individuals with MGD only, and aqueous-deficient individuals were ineligible for assignment to a study cohort. As a result, our study findings are applicable to individuals with and without MGD who are not aqueous deficient. Further studies are indicated to determine if visual performance in individuals with aqueous-deficient or mixed DED differs from that observed in our study in individuals with MGD.

## Conclusions

Tear film stability is important for the maintenance of visual performance. In this study, both BCVA and reading speed were reduced in individuals with severe MGD. For patients with MGD and visual complaints, reading speed testing in the office may be beneficial to detect patients’ difficulty with visual tasks in everyday life.
